# CRM1 Promotes Capsid Disassembly and Nuclear Envelope Translocation of Adenovirus Independently of Its Export Function

**DOI:** 10.1128/jvi.01273-21

**Published:** 2022-02-09

**Authors:** Floriane Lagadec, Irene Carlon-Andres, Jessica Ragues, Sarah Port, Harald Wodrich, Ralph H. Kehlenbach

**Affiliations:** a Department of Molecular Biology, Faculty of Medicine, Göttingen Center of Biosciences (GZMB), Georg-August-University Göttingen, Göttingen, Germany; b CNRS UMR 5234, Fundamental Microbiology and Pathogenicity, Université de Bordeaux, Bordeaux, France; International Centre for Genetic Engineering and Biotechnology

**Keywords:** adenovirus, CRM1, nuclear transport, capsid disassembly, genome delivery

## Abstract

After receptor-mediated endocytosis and endosomal escape, adenoviral capsids can travel via microtubule organizing centers to the nuclear envelope. Upon capsid disassembly, viral genome import into nuclei of interphase cells then occurs through nuclear pore complexes, involving the nucleoporins Nup214 and Nup358. Import also requires the activity of the classic nuclear export receptor CRM1, as it is blocked by the selective inhibitor leptomycin B. We have now used artificially enucleated as well as mitotic cells to analyze the role of an intact nucleus in different steps of the viral life cycle. In enucleated U2OS cells, viral capsids traveled to the microtubule organizing center, whereas their removal from this complex was blocked, suggesting that this step required nuclear factors. In mitotic cells, on the other hand, CRM1 promoted capsid disassembly and genome release, suggesting a role of this protein that does not require intact nuclear envelopes or nuclear pore complexes and is distinct from its function as a nuclear export receptor. Similar to enucleation, inhibition of CRM1 by leptomycin B also leads to an arrest of adenoviral capsids at the microtubule organizing center. In a small-scale screen using leptomycin B-resistant versions of CRM1, we identified a mutant, CRM1 W142A P143A, that is compromised with respect to adenoviral capsid disassembly in both interphase and mitotic cells. Strikingly, this mutant is capable of exporting cargo proteins out of the nucleus of living cells or digitonin-permeabilized cells, pointing to a role of the mutated region that is not directly linked to nuclear export.

**IMPORTANCE** A role of nucleoporins and of soluble transport factors in adenoviral genome import into the nucleus of infected cells in interphase has previously been established. The nuclear export receptor CRM1 promotes genome import, but its precise function is not known. Using enucleated and mitotic cells, we showed that CRM1 does not simply function by exporting a crucial factor out of the nucleus that would then trigger capsid disassembly and genome import. Instead, CRM1 has an export-independent role, a notion that is also supported by a mutant, CRM1 W142A P143A, which is export competent but deficient in viral capsid disassembly, in both interphase and mitotic cells.

## INTRODUCTION

Adenoviruses (Ads) are nonenveloped DNA viruses that infect dividing and nondividing cells. Their icosahedral capsid shell is composed of three major proteins, hexon, penton, and fiber, and minor proteins IIIA, VI, VIII, and IX. The capsid surrounds and protects the viral core, with the adenoviral genome highly compacted by the core proteins VII, V, and X and protected at each extremity by the covalently bound terminal protein TP (1, 2). Ads enter cells by receptor-mediated endocytosis (3). The uptake process induces structural changes in the capsid that release the membrane lytic internal capsid protein VI within the endosomal compartment (4–7). Protein VI contains an amphipathic helix that binds and mediates endosomal membrane lysis to permit viral escape (8–11). Cells recognize virus-induced membrane damage via cytosolic galectins (12, 13) that cluster at the site of membrane penetration and mount an autophagic response to remove the damaged endosome (14, 15). During the escape process, Ads delay the cell response to avoid degradation. This process gives Ads enough time to access the cytoplasm to engage in motor binding for microtubule-directed transport to the nucleus to deliver their genome (15–18). Despite the partial disassembly required for endosomal escape, Ads remain as physical entities during cytoplasmic transport, and several studies have shown that particles enrich at the level of the microtubule organizing center (MTOC) prior to their accumulation at the nuclear envelope (NE) (19–22). There, Ad particles dock via hexon at the nuclear pore complex (NPC) by binding Nup214 (23, 24), a nucleoporin that faces the cytoplasmic side of the NPC (25). NPC engagement is thought to trigger disassembly of the capsid at the level of the NPC, liberating the viral genome and priming it for nuclear import (26–28). Genome import into the nucleus is likely to involve the binding of nuclear transport receptors (NTRs) to protein VII, which contains several functional nuclear localization signals (NLS) and binds the genome at regular intervals (29–33). The cytoplasmic nucleoporin Nup358 (34) was recently shown to facilitate Ad genome import, probably by facilitated formation of transport complexes between the adenoviral genome and transport receptors (26). While protein VII can functionally interact with more than one NTR, several studies point to transportin-1 (TNPO1) as the most relevant import receptor for Ads (26, 30, 33). TNPO1 is a member of the family of importin β-like transport receptors and is known to import a number of cellular proteins into the nucleus (35, 36). Curiously, not only import receptors but also the classic nuclear export receptor chromosomal region maintenance 1 (CRM1; also known as exportin 1 [XPO1]) plays a role at early stages of infection, i.e., in nuclear transport of adenoviral capsids to the nuclear envelope and promoting adenovirus genome import into the nucleus (37, 38). Later, CRM1 may also be involved in nuclear export of early viral transcripts (39). Like TNPO1, CRM1 belongs to the importin β family and transports proteins containing a nuclear export signal (NES) out of the nucleus (40–44). The small GTP-binding protein Ran plays an important role in export as well, as RanGTP is required for the formation of the initial export complex (45). Several hundred cellular proteins have been identified as CRM1 cargoes, mostly by proteomic means (46, 47). CRM1 also interacts with certain nucleoporins, and we recently solved the structure of a fragment of Nup214 in a complex with CRM1, RanGTP, and snurportin 1 (SPN1) as an export cargo (48). The fungal metabolite leptomycin B (LMB) has been instrumental for studying CRM1-dependent nuclear export (49, 50). It covalently binds to a reactive cysteine residue (Cys528) in the NES-binding cleft of CRM1, strongly reducing the receptor-cargo interaction and thus inhibiting nuclear export (51, 52). In addition to its prominent function as a nuclear export receptor, CRM1 may also play a role independent of an intact nucleus, e.g., in mitosis. It binds, for example, to kinetochores and affects microtubule-dependent chromosome segregation during anaphase (53). CRM1 was also found at the MTOC, where it was suggested to interact with the centrosomal marker protein pericentrin (54). Furthermore, phosphorylation of CRM1 has been shown to promote the recruitment of Nup358 and RanGAP, the GTPase activating protein for Ran, to the mitotic spindle (55).

While there is consensus that in interphase (i.e., in cells with an intact NE) Ads depend on NPC binding for genome delivery, it remains unclear if a passage through the MTOC is also a requirement. Treating cells with LMB resulted in strong accumulation of Ads at the MTOC (37). This accumulation prevented subsequent NPC interaction and blocked genome liberation from the capsid. Expression of an LMB-resistant CRM1 mutant restored Ad genome delivery, showing that CRM1 is crucial for the translocation from the MTOC to the NE (38). The study suggested that CRM1 supports unloading of Ads from microtubules, affecting Ad trafficking at the vicinity of the NE (38). If CRM1 exerts this role directly or mediates the nuclear export of an essential factor driving the process is not yet known.

In this report, we confirm the essential role for CRM1 in Ad translocation from the MTOC to the NPC in U2OS cells. We identify a CRM1 mutant (W142A P143A) that is defective in the process but remains export competent. Furthermore, we find that Ads efficiently release their genome when infecting mitotic cells devoid of the NE. We show that genome release in mitotic cells is promoted by wild-type CRM1 but not by the CRM1 mutant, identifying a novel role for CRM1 in capsid disassembly and/or genome liberation that is distinct from its function as a nuclear export receptor.

## RESULTS

### Adenoviruses infect and traffic to the microtubule organizing center in the absence of a nucleus.

**FIG 1:**
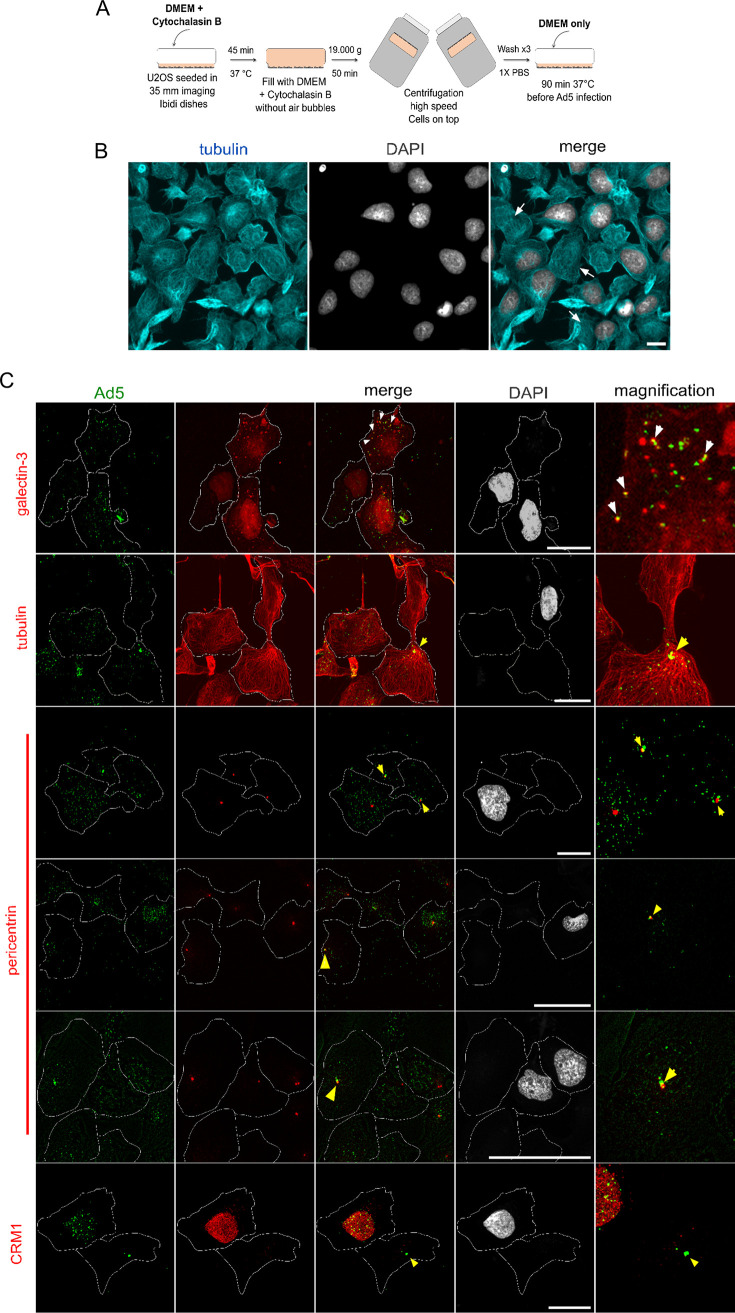
Nuclear factors are required to remove Ad5 from the MTOC. (A) Protocol for enucleation of U2OS cells (see the text for details). (B) After enucleation, cells were fixed, stained with anti-tubulin antibodies and DAPI, and imaged by fluorescence microscopy, showing a single plane. Arrows, cells lacking a nucleus but with intact microtubules. Scale bar, 50 μm. (C) U2OS cells constitutively expressing galectin-3 mCherry (top row) or U2OS cells were enucleated and infected for 2 h with Alexa 488-labeled Ad5 particles. Cells were fixed and directly analyzed for mCherry and Alexa 488 fluorescence (top row) or subjected to indirect immunofluorescence, with detection of tubulin, pericentrin, and CRM1, as indicated. DAPI staining (gray) was used to identify remaining nuclei. Cells were imaged by confocal microscopy, and maximal-projection images are shown. The cell periphery was manually drawn after increasing the contrast of the Alexa 488 channel image in Image J. White arrows, colocalization between galectin-3 and Ad5 capsids. Yellow arrowheads, accumulation of Ad5 in a cell lacking a nucleus. Scale bars, 20 μm. (D) U2OS cells were treated with (+LMB) or without (−LMB) LMB for 45 min. Infection with Alexa 488-labeled Ad5 particles was performed in the presence (+LMB) or absence (−LMB) of LMB for 2 h. After infection, cells were either left at 37°C or 4°C for 30 min or permeabilized with digitonin for 5 min at 37°C. After fixation, cells were stained with anti-tubulin (cyan) and antipericentrin (red) antibodies. Ad5 capsids were identified by the Alexa 488 fluorescence, and DNA was stained with DAPI (gray). Cells were imaged by fluorescence microscopy, and one plane is shown. Scale bars, 50 μm.

Ads enter cells through endocytosis, escape from the endosome, and use cytosolic microtubule-directed transport toward the nucleus (56). Studies in enucleated A549 human lung epithelial cells showed that Ad species serotype 5 (Ad5) capsids accumulate at the MTOC after infection (57). To confirm that this process occurs through natural infection without the need for a nucleus and/or nuclear factors, we adapted enucleation to U2OS cells followed by infection with Ad5 capsids (Fig. 1). U2OS cells support the entire Ad5 life cycle, are not virally transformed, and are easy to image. We treated U2OS cells with cytochalasin B to depolymerize actin filaments (58). Nuclei were then mechanically removed by high-speed inverted centrifugation of cells grown on dishes, followed by a 90-min recovery time in Dulbecco’s modified Eagle medium (DMEM) (Fig. 1A). After recovery, we added fluorescent antibodies to the medium and verified cell integrity by fluorescence exclusion from the enucleated cell lumen (see Movie S1 in the supplemental material). Staining enucleated cells with tubulin-specific antibodies and DAPI (4′,6-diamidino-2-phenylindole) showed that they retained the microtubule network and that the efficiency of enucleation was ∼50% (Fig. 1B). Nonenucleated cells in the same sample were subsequently used as internal controls. Ad5 endosome lysis is conditional of controlled postentry disassembly and results in galectin-3 (Gal3) accumulation on endosome penetration sites (14, 59). Therefore, we used Gal3-mCherry-expressing cells for the enucleation experiment and infected the treated cells with fluorescently labeled Ad5 particles to verify that they enter by receptor-mediated endocytosis and escape from the endosome by endosome lysis. We observed colocalization of viral particles with Gal3 in cells containing a nucleus as well as in enucleated cells (Fig. 1C, arrows). In enucleated cells, Ad5 particles accumulated in a specific spot, associated with converging microtubules and identified as the MTOC by pericentrin costaining. This association was quite stable, and we followed it up to 2 h postinfection (p.i.), but it can last even longer (57). In control cells containing a nucleus, in contrast, Ad5 particles were distributed more evenly around the nucleus, without specific MTOC accumulation (Fig. 1C). Cytochalasin B treatment did not change the apparent tubulin organization, and nuclear factors such as CRM1 were largely removed in enucleated cells (Fig. 1C). The MTOC retention of Ad5 resulting from LMB treatment (37) was also maintained after microtubule depolymerization induced by permeabilizing the cells with digitonin, i.e., in the absence of cytosolic components, as well as after microtubule depolymerization induced by cold treatment (Fig. 1D). These results showed that stable accumulation of Ad5 at the MTOC is independent of microtubule integrity and soluble cytosolic factors. Our data confirm that Ad5 cell entry proceeds normally in enucleated cells and does not require nuclear factors. The MTOC is an endpoint of the process, and the long-term stability of the Ad5 association (5) in enucleated cells suggests that nuclear factors are required for removal of viral particles from the MTOC.

### Pharmacological inhibition of CRM1 arrests Ad5 at the MTOC, preventing genome delivery.

**FIG 2:**
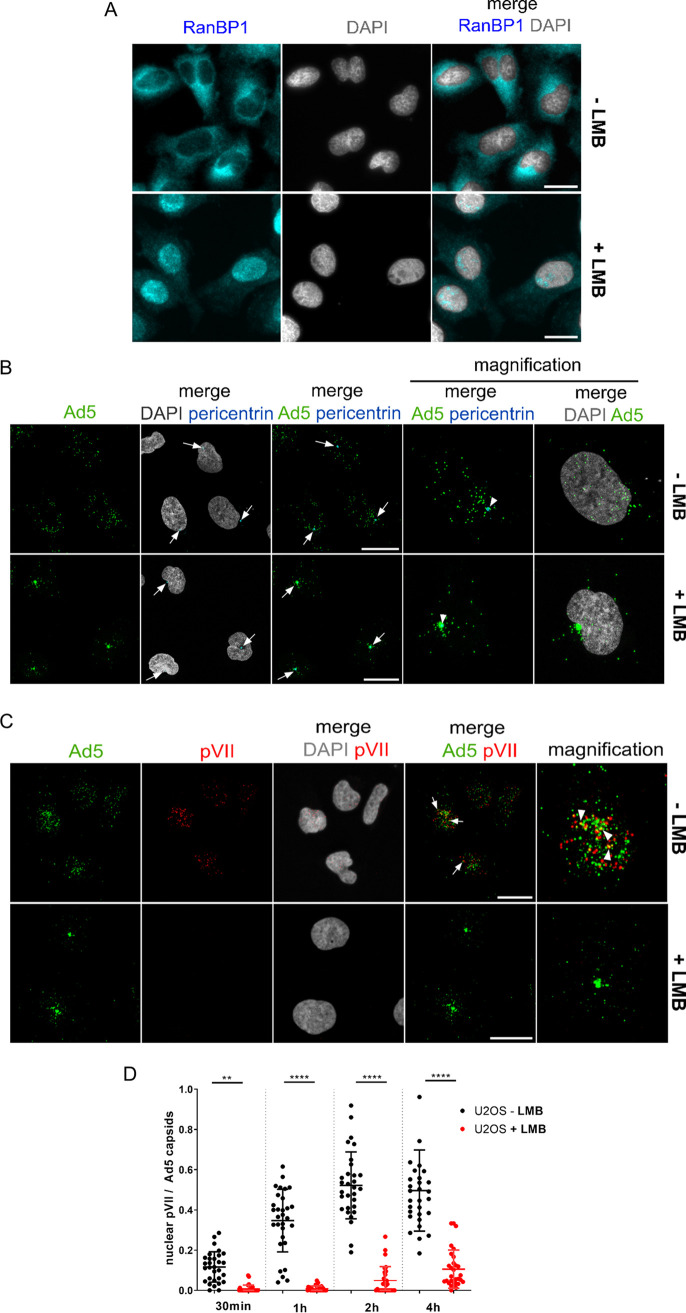
Inhibition of CRM1 blocks Ad5 capsids at the MTOC and impairs genome delivery. U2OS cells were treated with (+) or without (−) LMB for 45 min, as indicated, and infected (B, C, and D) or not (A) with Alexa 488-labeled Ad5. (A) Cells were subjected to indirect immunofluorescence detecting RanBP1 (cyan) and chromatin (DAPI; gray). Scale bars, 20 μm. (B) Cells were infected for 1 h, fixed, and subjected to indirect immunofluorescence detecting pericentrin (cyan) and chromatin (DAPI; gray). Arrows, MTOCs, as detected by pericentrin staining. Scale bars, 20 μm. (C) Cells were infected for 1 h, fixed, and subjected to indirect immunofluorescence detecting Ad5 capsids (green), pVII (red), and DNA (DAPI; gray). Cells were imaged by confocal microscopy, and maximal-projection images are shown. Arrows, colocalization events between Ad5 capsids and pVII. Scale bars, 20 μm. (D) Cells were infected for up to 4 h as indicated and analyzed as described for panel C. The scatterplot shows the quantification over time of the total number of nuclear pVII foci, normalized to the total number of Ad5 capsids, in the absence (black dots) or presence (red dots) of LMB. Mean values (±SD) of 30 cells per condition are shown. Statistical analysis was performed using a one-way ANOVA multicomparison test.

Treating cell lines with the CRM1 inhibitor leptomycin B (LMB) arrests Ad5 at the MTOC (37), resulting in a phenotype comparable to what we observed in enucleated cells. We thus repeated this assay in our model U2OS cells. Cells were preincubated with 20 nM LMB and infected with Ad5 in the continued presence of LMB. LMB treatment specifically impairs binding between CRM1 and NES-containing cargo proteins (51, 52). Nuclear retention of RanBP1, a known export cargo of CRM1 (60), was used as a control for the LMB effect (Fig. 2A). Infected U2OS cells treated with LMB showed a strong accumulation of Ad5 capsids at the MTOC, as revealed by costaining for pericentrin (Fig. 2B) that lasted for up to 8 h. We next performed an infection time course in the absence or presence of LMB and quantified nuclear genome delivery over time. Nuclear Ad5 genome delivery can be monitored by staining for the genome-bound protein VII (pVII) (61). In this assay, single nuclear genomes bound to ∼500 copies of pVII can be identified as dots after the Ad5 capsid has docked at the nuclear envelope and genome import has occurred (26) (Fig. 2C). Quantification of nuclear genomes under control conditions revealed rapid viral genome import starting at ∼30 min p.i. and reaching a plateau at ∼2 h p.i. In the presence of LMB, genome import was severely impaired (Fig. 2D) and the amount of observable pVII signal was reduced, even after 4 h p.i. Moreover, capsids trapped at the MTOC did not release genomes, since no pVII signal was detectable, neither in the nucleus nor at the MTOC area nor elsewhere in the cytoplasm. These results confirm that Ad5 requires functional CRM1 to be translocated from the MTOC to the nuclear envelope. Whether CRM1 function is required only for capsid translocation or also for genome release is not known.

### CRM1 mutant W142A P143A is compromised in Ad genome delivery.

**FIG 3:**
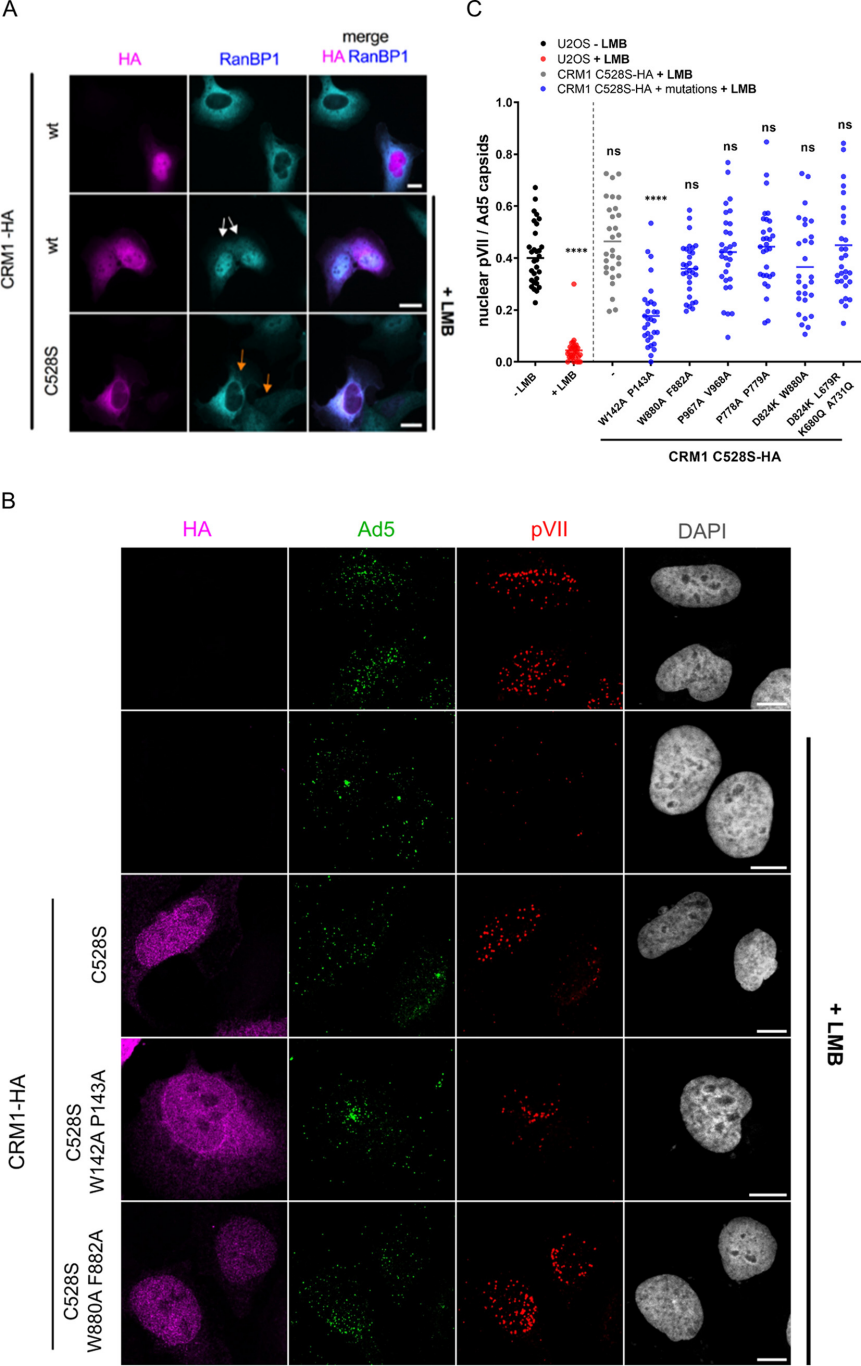
A CRM1 mutant (W142A P143A) is compromised in promoting Ad5 genome import. U2OS cells were left untransfected or were transfected with versions of CRM1-HA C528S as indicated and treated with (+) or without (−) LMB for 45 min. Cells were infected (B and C) or not (A) for 1 h with Ad5 particles, in the presence (+LMB) or absence (−LMB) of LMB. (A) Cells were subjected to indirect immunofluorescence detecting CRM1-HA wild type (wt) or C528S mutant (magenta), RanBP1 (cyan), and chromatin (DAPI; gray). White arrows, CRM1-HA wt-transfected cells treated with LMB, showing nuclear retention of RanBP1. Orange arrows, CRM1-HA C528S-transfected cells treated with LMB, showing rescued export of RanBP1. Scale bars, 20 μm. (B) Cells were fixed and stained with anti-HA (magenta), anti-Ad5 capsids (green), and anti-pVII (red) antibodies and imaged by confocal microscopy. Maximal-projection images are shown for three of the CRM1-HA constructs. Scale bars, 20 μm. (C) Cells were treated as described for panel B, and the number of nuclear pVII dots, normalized to the total number of capsids, was analyzed. CRM1 mutations are indicated below the *x* axis of the scatterplot. Mean values (±SD) of 30 cells per condition are shown. Statistical analysis was performed using a one-way ANOVA multicomparison test and Tukey's multiple comparison post hoc test.

To show that the LMB effect on genome import is a consequence of CRM1 inhibition and not an indirect effect, we performed a rescue experiment using an LMB-resistant CRM1 mutant. In this mutant, cysteine 528 (the target of LMB) was mutated to a serine (C528S), preventing covalent binding of LMB to CRM1 (51). Functional rescue in cells overexpressing hemagglutinin (HA)-tagged CRM1 C528S but not the wild-type protein was verified by restoring export of endogenous RanBP1 in the presence of LMB (Fig. 3A). We next infected U2OS cells with Ad5 in the presence or absence of LMB (Fig. 3B). Ad5 genome delivery was detected by pVII staining. Overexpression of CRM1 C528S-HA relieved Ad5 accumulation at the MTOC and restored nuclear genome import, confirming that CRM1 is the LMB target controlling genome import as previously reported (38).

Previous reports suggested association of Ad5 capsids with the nuclear pore through Nup214 (23, 24). To identify potential functional domains in CRM1 outside the NES binding site, we repeated the rescue assay using a set of CRM1 constructs with mutations in close proximity of some identified binding sites of Nup214 available from a previous study (48). All mutants were cloned as versions of LMB-resistant CRM1 C528S-HA. LMB-treated U2OS cells were transfected with these constructs or the CRM1 C528S-HA control and infected with Ad5. Staining for pVII suggested that several of the mutants were able to rescue genome import, similar to the original C528S-HA version (Fig. 3B). A quantification of nuclear pVII for all tested mutants is shown in Fig. 3C. All CRM1 mutants except one were able to rescue genome import to wild-type control levels, suggesting that they did not impact the CRM1 function needed during Ad5 infection. CRM1 W142A P143A C528S was the only mutant that was severely compromised in its ability to prevent Ad5 accumulation at the MTOC and rescue genome import. Therefore, we decided to characterize this mutant in detail.

### Biochemical and functional characterization of purified CRM1 W142A P143A C528S.

**FIG 4:**
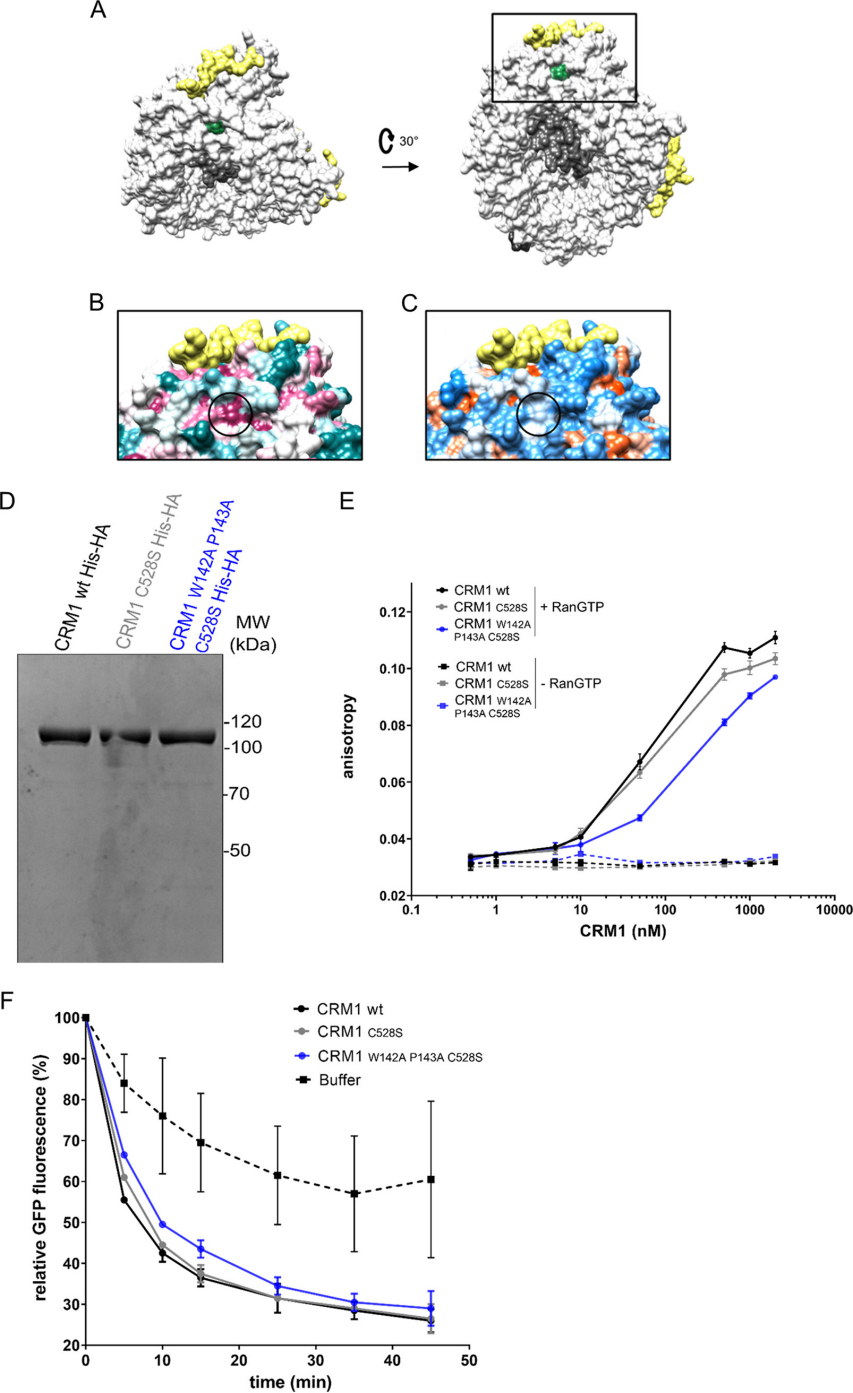
Purified CRM1 W142A P143A promotes nuclear protein export in permeabilized cells. (A) Crystal structure of the Nup214-CRM1 export complex (PDB ID 5DIS) in surface representation. CRM1 is depicted in white with amino acid residues 142 and 143 highlighted in green (Nup214, yellow; RanGTP, gray; SPN1, black). The view in the right panel corresponds to a 30° rotation along the *x* axis. (B and C) Enlarged view of the boxed region in the right portion of panel A. The circle surrounds CRM1 amino acid residues 142 and 143. Nup214 is depicted in yellow. (B) CRM1 was colored by conservation using ConSurf with a gradient from magenta (conserved) to teal (not conserved). (C) CRM1 was colored using the Chimera hydrophobicity surface preset with hydrophobic regions in white and charged residues in blue and red. (D) Tagged versions of wild-type and mutant CRM1 were purified from bacteria and analyzed by SDS-PAGE, followed by Coomassie staining. (E) A 6-carboxyfluorescein-labeled peptide derived from the NES of PKI was incubated in the presence or absence of RanQ69L-GTP 1–180 and increasing concentrations of different versions of purified wild-type or mutant CRM1-HA, as indicated. Fluorescence polarization was measured, and anisotropy was plotted as the mean of two independent experiments (only one experiment for the −RanQ69L-GTP control). (F) HeLa cells expressing GFP-NFAT were permeabilized with digitonin and subjected to nuclear export reactions in the presence of Ran and buffer or Ran and versions of purified wild-type or mutant CRM1, as indicated. After reactions for up to 45 min, residual fluorescence was measured by flow cytometry. The graph shows the variation from the mean of two independent experiments.

The localization of the CRM1 W142A P143A mutation identified in our small-scale screen is highlighted in our previously published crystal structure of the CRM1-SPN1-Ran-Nup214 complex (48) (Fig. 4A to C). CRM1 amino acid residues 142 and 143 (green) are positioned at the N terminus of CRM1 (white), which forms a ring-like structure enclosing RanGTP (gray) and is stabilized by the binding of the Nup214 FG region (yellow) to its N- and C-terminal regions (Fig. 4A). The identified residues are located in the rough vicinity of RanGTP and the Nup214 fragment but not close enough to interact with either of these two proteins. The NES-binding cleft on CRM1 with the bound cargo SPN1 is located on the opposite side of the CRM1 ring. CRM1 amino acid residues 142 and 143 are positioned in the center of a conserved (Fig. 4B) and hydrophobic (Fig. 4C) patch on CRM1, suggesting that they are of functional relevance. Therefore, we investigated the possibility that the mutation affects the pertinent biochemical properties and/or the general export functions of CRM1. First, we purified wild-type CRM1 and the C528S and W142A P143A C528S mutants from bacteria (Fig. 4D). We then used an established assay based on fluorescence polarization to monitor complex formation between a fluorescently labeled NES peptide and our CRM1 versions (62). Upon complex formation, which requires the presence of RanGTP, the rotational mobility of the labeled peptide is decreased, leading to changes in fluorescence polarization. As shown in Fig. 4E, the addition of increasing concentrations of wild-type CRM1 resulted in higher levels of anisotropy, reflecting complex formation. The introduction of the C528S mutation had only a very small effect, whereas the mutant containing the additional W142A P143A mutation exhibited a somewhat reduced affinity for the NES peptide. In the absence of RanGTP, no changes in anisotropy levels were observed, demonstrating the specificity of the assay.

Next, we used the purified CRM1 versions with respect to their ability to promote nuclear protein export, taking advantage of an established *in vitro* system. This assay is based on nuclear export of green fluorescent protein (GFP)-NFAT in stably transfected cells (41). Briefly, cells with nuclear GFP-NFAT are permeabilized with digitonin, leading to the release of relevant transport factors, including Ran and CRM1. These proteins can then be added back to the reaction to initiate nuclear export. After the reaction, the residual nuclear fluorescence is measured by flow cytometry, allowing the analysis of a large number of cells in a short period of time. As shown in Fig. 4F, in the presence of Ran alone, the cells lost up to 40% of their initial fluorescence over the course of the experiment, probably reflecting the presence of some residual endogenous CRM1 in the permeabilized cells. The addition of wild-type CRM1 or the two mutant versions of the export factor then further promoted nuclear export of GFP-NFAT to similar extents. Together, these results show that the W142A P143A mutation in CRM1 does not lead to an obvious defect in nuclear protein export *in vitro*, although our biochemical analyses suggest a modest reduction in the affinity for export cargoes.

### CRM1 W142A P143A C528S is functional in living cells.

**FIG 5:**
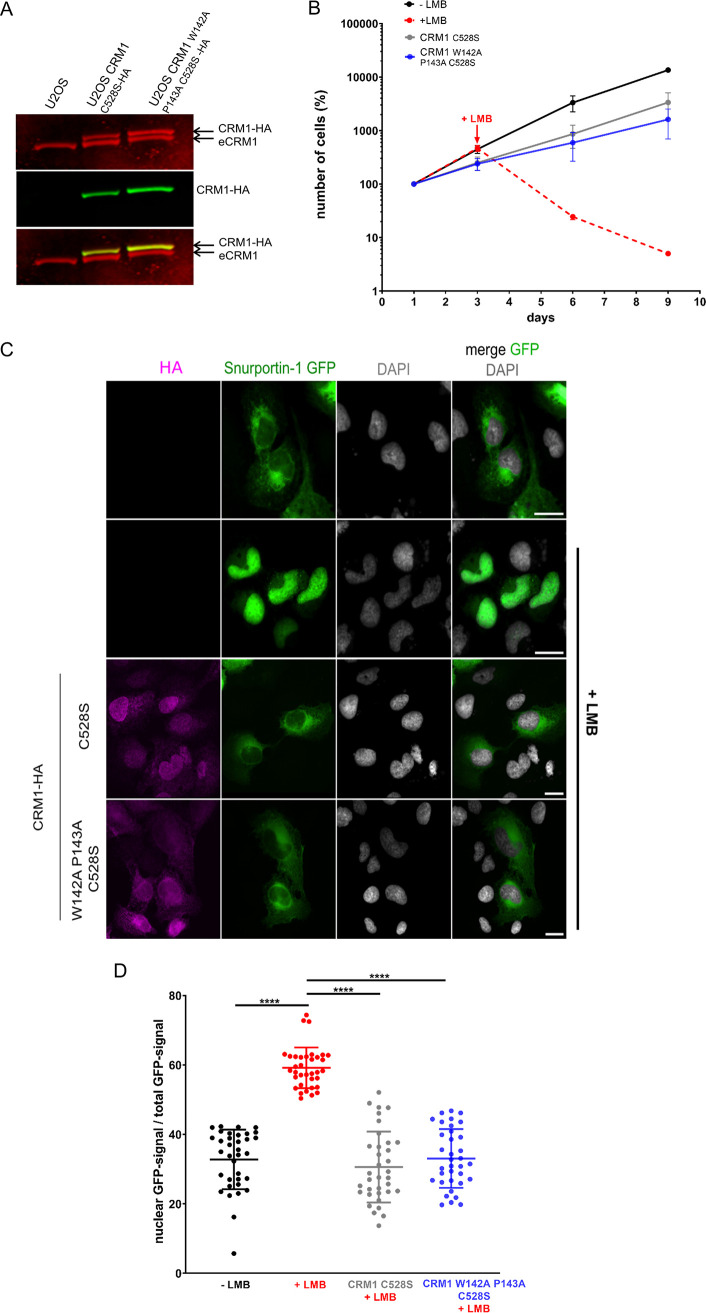
The CRM1-dependent export pathway is not compromised in U2OS cells constitutively expressing CRM1 W142A P143A. (A) Detection of endogenous (eCRM1) and overexpressed (CRM1-HA) CRM1 in stably transfected U2OS cells by Western blotting using antibodies against CRM1 (red) or the HA tag (green). The merge of the two signals is shown in the lower panel. (B) Growth analysis of U2OS cells constitutively expressing CRM1-HA. Cells were counted over a course of 9 days (200 h), and the number of cells was plotted as the percentage of the starting value on day 1. Black curve, wild-type cells, no LMB; red curve, wild-type cells with 2 nM LMB added on day 3; gray (CRM1 C528S-HA) and blue (CRM1 W142A P143A C528S-HA) curves, cells were maintained from day 1 in 2 nM LMB. Error bars represent the variation from the mean of two independent experiments. (C) Wild-type U2OS cells or U2OS cells stably expressing CRM1 C528S-HA or CRM1 W142A P143A C528S-HA were transfected with a construct coding for snurportin-1 GFP (SNP-1). At 24 h posttransfection, LMB was added (+LMB) to wild-type cells for 45 min as indicated. CRM1-HA-expressing cells were constantly kept in medium containing 2 nM LMB. Cells were fixed and analyzed by fluorescence microscopy, detecting CRM1-HA (magenta), GFP-SNP-1 (green), or DNA (DAPI; gray). Scale bars, 20 μm. (D) Quantitative analysis of the results in panel C, comparing the nuclear GFP signal/total GFP signal ratios. The quantification from a single experiment was performed on maximal-projection images of 35 cells.

To investigate the functionality of our CRM1 mutant *in vivo*, we first generated stable U2OS cells expressing either the CRM1 C528S single mutant or the CRM1 W142A P143A C528S triple mutant, both with a C-terminal HA tag to facilitate detection. Selection of cells was based on their resistance to LMB, as conferred by the cysteine-to-serine mutation. As shown in the Western blot in Fig. 5A, the expression level of exogenous CRM1 was very similar to that of the endogenous protein in both selected cell populations. As a first characterization, we then analyzed the growth of the different cells in the absence or presence of LMB. Wild-type U2OS cells grew with a doubling time of ∼33 h. After the addition of LMB, these cells stopped dividing and died during the course of the experiment (Fig. 5B). Cells expressing exogenous CRM1 were kept in LMB-containing medium at all times to inhibit endogenous CRM1. Compared to wild-type cells in the absence of LMB, they grew somewhat slower. Cells expressing the CRM1 W142A P143A C528S triple mutant, however, had a growth rate very similar to that of cells expressing CRM1 C528S, suggesting that the additional mutations at positions 142 and 143 do not impose a disadvantage with respect to viability and cell division. Importantly, both cell types were viable in the presence of LMB and showed exponential growth. Next, we specifically analyzed the ability of the mutant versions of CRM1 to promote nuclear protein export *in vivo*. Wild-type U2OS cells or cells expressing exogenous versions of CRM1 were transiently transfected to express SPN1-GFP, an established CRM1 export cargo. In the absence of LMB, the majority of this reporter protein was found in the cytoplasm of control cells, whereas it accumulated in the nucleus upon addition of the drug (Fig. 5C and D). In cells expressing either the CRM1 C528S single mutant or the CRM1 W142A P143A C528S triple mutant, in contrast, SPN1-GFP was largely cytoplasmic, even in the presence of LMB. This result shows that the mutations at positions 142 and 143 (W142A P143A) do not affect the ability of CRM1 to promote nuclear export of a cargo protein. Together, our data suggest that the defect in adenoviral genome import as observed for the W142A P143A mutant of CRM1 is not a direct consequence of reduced CRM1-dependent nuclear protein export.

### Cells expressing CRM1 W142A P143A C528S delay Ad5 genome delivery.

**FIG 6:**
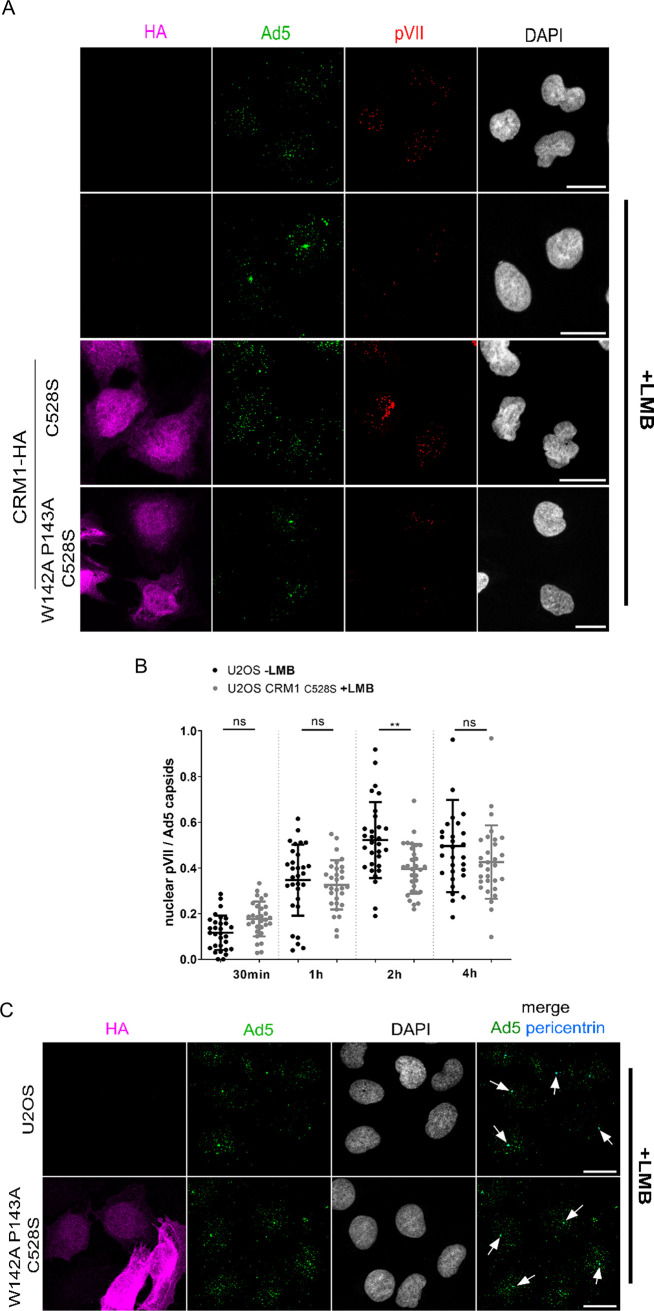
CRM1 W142A P143A delays the first steps of Ad5 infection. (A to C) Wild-type U2OS cells or U2OS cells expressing CRM1-HA were infected with Ad5-GFP particles for 30 min to 4 h in the absence or presence (+) of LMB. Cells were fixed and stained with anti-HA (magenta), anti-Ad5 capsids (green), anti-pVII (red), or anti-pericentrin (blue) antibodies and with DAPI (gray) for chromatin staining. (A) Confocal images of cells after 2 h of infection. Maximal-projection images are shown. Scale bars, 20 μm. (B and D) Scatterplots showing quantifications of the total number of pVII foci colocalizing with DAPI signal per cell, normalized to the total number of Ad5 capsids. Quantifications of U2OS cells not treated with LMB are depicted in black, those of U2OS cells expressing CRM1 C528S-HA cells are in gray, and those of U2OS cells expressing CRM1 W142A P143A C528S-HA cells are in blue. Mean values (±SD) of 30 cells per condition are shown. Statistical analysis was performed using a one-way ANOVA multicomparison test. (C) Confocal images of cells after 2 h of infection. Maximal-projection images are shown. White arrows, pericentrin positions and/or colocalization events between pericentrin and Ad5 capsids. Scale bars, 20 μm. (E and F) Wild-type U2OS cells or U2OS cells expressing CRM1-HA were infected with Ad5-GFP particles for 2 to 6 h in the absence or presence (+) of LMB. Cells were fixed, and E1A transcripts (magenta) were detected using specific RNA probes (RNAScope). A second staining was used, and cells were stained with anti-Ad5 capsids (green) antibodies and with DAPI (gray) for chromatin staining. (E) Confocal images of cells after 6 h of infection. Maximal-projection images are shown. Scale bars, 20 μm. (F) Scatterplot showing the quantification of the total number of E1A foci per cell. Quantifications of U2OS cells expressing CRM1 C528S-HA are depicted in gray, and those of U2OS cells expressing CRM1 W142A P143A C528S-HA are depicted in blue. Mean values (±SD) of 30 cells per condition are shown. Statistical analysis was performed using a one-way ANOVA multicomparison test. (G) Plaque assays were performed on wild-type U2OS cells (black) or U2OS cells expressing CRM1 C528S-HA (gray) or CRM1 W142A P143A C528S-HA (blue). A 2 nM concentration of LMB was added to CRM1-HA-expressing cells as indicated. At 6 days postinfection, plaques were counted using bright-field microscopy and the total number of plaques per condition was plotted. Error bars depict the standard deviation from the mean of two independent experiments.

Our analysis showed that U2OS cells stably expressing CRM1 C528S or CRM1 W142A P143A C528S behaved normally in terms of cargo export. We next addressed the question of whether the two cell types differed with respect to Ad5 infection. U2OS cells were infected and fixed at different time points, and the number of nuclear genomes was monitored by quantifying nuclear pVII dots over time (Fig. 6). At 2 h p.i., LMB-treated control U2OS cells accumulated Ad5 at the MTOC, which was not observed in stably CRM1 C528S-HA-expressing cells or untreated control cells (Fig. 6A). Nuclear import of viral genomes occurred rapidly starting at 30 min p.i. and with similar efficiencies in CRM1 C528S-HA rescue cells and control cells that had not been treated with LMB (Fig. 6B). In contrast, in cells stably expressing CRM1 W142A P143A C528S-HA, Ad5 capsids accumulated at early time points (2 h p.i.) to levels at the MTOC similar to those observed in U2OS control cells treated with LMB (Fig. 6A), as verified by costaining with pericentrin (Fig. 6C). When we compared cells expressing CRM1 W142A P143A C528S-HA or CRM1 C528S-HA with respect to nuclear genome accumulation over time, we noted that nuclear pVII dots in the triple mutant cells did not accumulate until 2 h p.i., followed by a slow but steady increase (Fig. 6D). This result showed that cells constitutively expressing CRM1 W142A P143A C528S displayed a phenotype similar to that seen in LMB-treated cells, resulting in a severe time delay in genome import. As a consequence, the onset of viral gene expression was also delayed, as verified by the analysis of E1A expression, using direct detection and quantification of E1A transcripts over time by *in situ* hybridization using RNAScope technology (63) (Fig. 6E). The level of E1A transcripts 6 h p.i. was significantly lower in cells constitutively expressing CRM1 W142A P143A C528S (Fig. 6F). To investigate if the CRM1 mutation affected overall viral infectivity, we next monitored Ad5 infection in U2OS CRM1-HA cells by plaque assay (Fig. 6G). This assay measures the propagation of virus over several rounds of infection, and any defect affecting viral fitness should result in reduced plaque size and/or numbers. U2OS control cells without LMB and cells stably expressing the stable CRM1-HA mutants in the presence of LMB were infected with different multiplicities of infection (MOI of 1, 0.1, and 0.01). At 6 days p.i., the total number of plaques per condition was counted. While U2OS control cells showed slightly higher numbers of plaques, probably due to a higher growth rate of the cells (see Fig. 5B), we did not detect any difference between the two other cell types, suggesting that a delay in genome delivery due to the W142A P143A mutation in CRM1 does not impact the overall virus replicative fitness.

### The nuclear envelope but not CRM1 is dispensable for Ad5 capsid disassembly and genome liberation.

**FIG 7:**
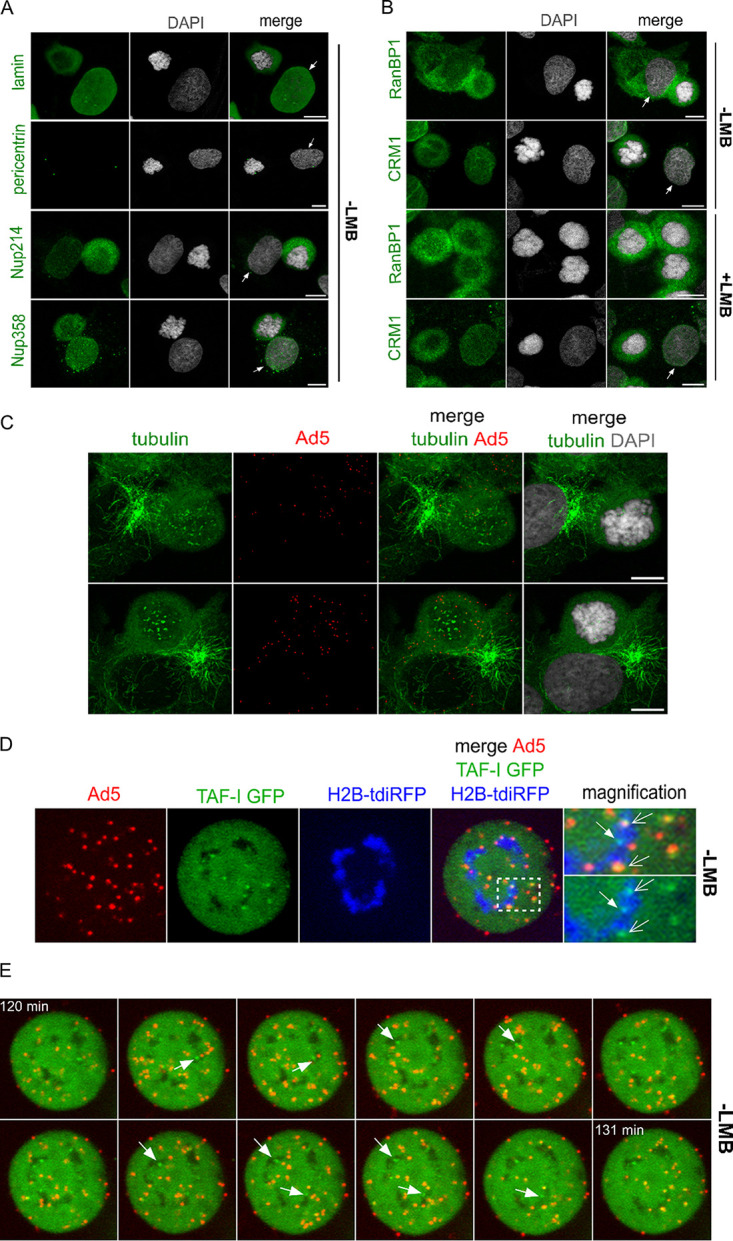
An intact nuclear envelope is dispensable for Ad5 capsid disassembly. (A and B) U2OS cells were synchronized in mitosis and treated with (+LMB) or without (−LMB) LMB for 45 min. Cells were fixed and subjected to indirect immunofluorescence using antibodies against lamin A/C, pericentrin, Nup214, and Nup358 (A) or CRM1 and RanBP1 antibodies (B). DAPI staining (gray) was used to identify chromatin. Cells were analyzed by confocal microscopy. White arrows, cells in interphase. Scale bars, 10 μm. (C) U2OS cells were synchronized in mitosis, infected for 1 h in the absence of Colcemid, fixed, and subjected to indirect immunofluorescence using antibodies against tubulin and Ad5 capsids (red). Cells were analyzed by confocal microscopy. Scale bars, 10 μm. (D to G) U2OS cells stably expressing TAF-I GFP were transfected with a construct coding for H2B-tdiRFP (blue) to stain chromatin. After 24 h, cells were synchronized in mitosis, treated (+LMB) or not (−LMB) with LMB, and infected with Alexa 594-labeled Ad5 particles. Mitotic cells were identified according to their chromatin staining (blue). Ad5 capsids (red) and Ad5 genomes (discrete TAF-I GFP dots; green) are shown. Living cells were imaged by spinning-disk confocal microscopy, and maximal-projection images are shown. (D and F) A mitotic U2OS TAF-I GFP cell at 130 min p.i., treated (+LMB) or not (−LMB) with LMB. TAF-I GFP dots without colocalizing Ad5 are marked with solid white arrows, whereas TAF-I GFP dots colocalizing with Ad5 are marked with open white arrows. (E and G) Overlay of TAF-I GFP (green) and Ad5 capsids (red) signals in a single mitotic cell over time, treated (+LMB) or not (−LMB) with LMB (starting at 120 min p.i. with 1-min intervals). TAF-I GFP dots with no colocalizing Ad5 capsids are marked with white arrows. (H and I) U2OS cells were synchronized in mitosis and treated with (+LMB) or without (−LMB) LMB for 45 min, followed by infection with Ad5 particles for 30 min, 1 h, or 2 h. (H) Cells were fixed and subjected to indirect immunofluorescence using antibodies against Ad5 capsids (green) and pVII (red). DAPI (gray) was used for chromatin staining. Colocalization events between Ad5 capsids and pVII are marked with white arrows. Cells were imaged by confocal microscopy, and maximal-projection images are shown. Scale bars, 10 μm. (I) Scatterplot showing the quantification of the total number of pVII foci per cell, normalized to the total number of Ad5 capsids, in the absence (black dots) or presence (red dots) of LMB. Mean values (±SD) of 30 cells per condition are shown. Statistical analysis was performed using a one-way ANOVA multicomparison test.

The NPCs play a central role in Ad5 genome delivery, essentially dividing the process into three conditional steps: (i) capsid docking at the cytosolic side of the NPC, (ii) capsid disassembly and genome liberation, and (iii) nuclear import of the viral genome. To further address the role of CRM1 in genome delivery, we thought to bypass the physical barrier of the NE/NPC and devised a protocol for Ad5 infection of mitotic cells. The advantage of this approach is that components of the NE and NPCs should be available in the cell but not in the physiological context of an intact nucleus. Our rationale was that detection of pVII upon direct infection of mitotic cells is the result of capsid disassembly, and no longer relies on the import step, because the NE barrier is absent. Cells were synchronized in mitosis with reversible Colcemid treatment, which blocked cells in metaphase due to its depolymerization effect on microtubules. Mitotic cells were identified via condensed chromosomes and their overall round shape. Arrested cells were stained with antibodies against nuclear lamins to verify the absence of the NE, against pericentrin to verify that the MTOCs were still intact, and against two major cytoplasmic nucleoporins, Nup214 and Nup358, to verify that they had entered a soluble intracellular pool and were no longer integrated into the NE (Fig. 7A). We next verified that CRM1 as well as the CRM1 cargo RanBP1 lost compartmentalization in mitosis and that treatment of cells with LMB did not affect their localization. As anticipated, CRM1 and its cargo both localized diffusely, irrespective of the LMB treatment (Fig. 7B). Next, we carried out Ad5 infections of mitotic cells in the presence or absence of LMB. Infections were performed in Colcemid-free medium, and cells were analyzed for up to 2 h p.i. before the cell cycle resumed due to the reversibility of the Colcemid block. To study the infection process in mitotic cells, we first controlled the state of microtubules after Colcemid treatment in infected U2OS cells (Fig. 7C). The tubulin staining indicated that no microtubules remained intact at least 2 h after Colcemid treatment. This result suggested that an intact microtubule network is dispensable for the first steps of Ad5 infection. We next used a live-cell imaging system based on cells stably expressing U2OS TAF-Iβ GFP and transfected with an expression vector for fluorescent histones (tdiRFP-H2B) to mark chromosomes. TAF-Iβ is a cellular factor known to form ternary complexes with pVII on incoming genomes (64) and can be used to fluorescently label incoming viral genomes in living cells (61). Cells were infected with fluorescently labeled Ad5 in the presence or absence of LMB, and nuclear midsections were imaged using spinning-disk confocal microscopy. The basal level of TAF-Iβ dots in noninfected cells was controlled (see Movie S4 in the supplemental material), and upon infection, TAF-Iβ GFP dots became apparent at approximately 1.5 to 2 h p.i. (Fig. 7D). Somewhat surprisingly, we readily observed TAF-Iβ dots well separated from capsids (Fig. 7D, solid arrows) as well as associated with capsids (Fig. 7D, open arrows). Moreover, virtually all free TAF-Iβ dots and several TAF-Iβ dots colocalizing with Ad5 capsids had restricted mobility and appeared associated with cellular chromatin, implying that the genomes became released and stably anchored to the chromatin (Fig. 7D, detail). Furthermore, the number of capsid-free TAF-Iβ dots increased over time, suggesting an active process (Fig. 7E; Movie S2). In cells treated with LMB, TAF-Iβ dots were also detectable. However, TAF-Iβ dots were far less abundant, almost exclusively associated with capsids (Fig. 7F, yellow signals; Movie S3), and only occasionally separated from capsids. However, chromatin targeting of TAF-Iβ-positive capsids was also observed (Fig. 7G). Quite unexpectedly, our live-cell imaging results showed that capsid disassembly and genome separation in mitotic cells are possible and do not require an intact NE with assembled NPCs nor an intact microtubule network. Moreover, once freed or partially liberated from capsids, genomes were targeted to chromatin, even in the presence of LMB, suggesting that the level of exposure was sufficient to make chromatin association possible. The strong reduction of free or capsid-associated TAF-Iβ dots observed upon LMB treatment suggested that CRM1 might be involved in Ad5 genome release from capsids. Because some noninfected mitotic control cells also showed a reduced number of TAF-1β dots (Movie S4), we wanted to confirm our observation in a system relying on endogenous proteins. We next repeated the mitotic cell infection assay in unmodified U2OS control cells in the presence or absence of LMB and stained for pVII in fixed mitotic cells to mark viral genomes (Fig. 7H). Antibody detection of individual separated pVII dots confirmed that genomes are efficiently released upon infection of mitotic cells and that this process does not require an intact NE. In several cases, capsids still colocalized with pVII signals, suggesting that we observed capsid disassembly (Fig. 7H, arrows). In contrast, upon LMB treatment, very few pVII signals were detectable. To quantify genome release in mitotic cells upon infection, we performed a time course analysis and determined the number of released genomes over time (Fig. 7I). This analysis confirmed that genome release was efficient and initiated within 1 h p.i. in mitotic control cells while genome delivery was severely suppressed in LMB-treated cells (Fig. 7I).

### CRM1 is a rate-limiting factor driving capsid disassembly and viral genome release independent of genome import.

**FIG 8:**
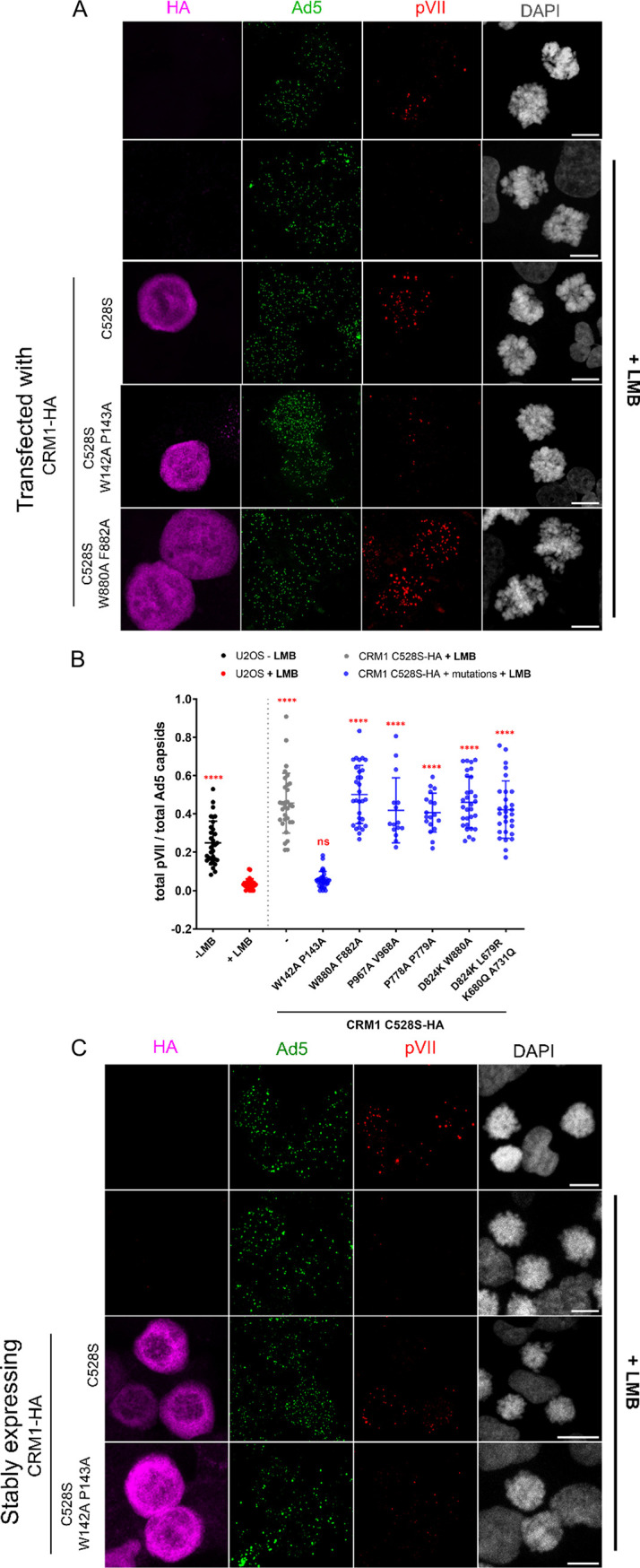
W142A P143A mutation in CRM1 induces a delay in Ad5 capsid disassembly in mitotic cells. (A and B) U2OS cells were left untransfected or were transfected with versions of CRM1-HA C528S as indicated below the *x* axis and treated with (+LMB) or without (−LMB) LMB for 45 min. Cells were synchronized in mitosis and infected for 1 h with Ad5 particles in the continued presence (+LMB) or absence of LMB. Cells were fixed and subjected to indirect immunofluorescence, with detection of CRM1-HA (magenta), Ad5 capsids (green), and Ad5 genomes (red) using antibodies against the HA tag, Ad5 capsids, and pVII, respectively. DAPI was used for chromatin staining. (A) Cells were imaged by confocal microscopy. Scale bars, 10 μm. (B) Quantification of the results shown in panel A. The scatterplot shows the number of pVII foci normalized to the number of Ad5 capsids per cell as detected on maximal-projection images. Mean values (±SD) of 30 cells per condition are shown. Statistical analysis was performed using a one-way ANOVA multicomparison test, comparing every condition to untransfected LMB-treated U2OS cells (red condition on the graph). (C to E) Wild-type U2OS cells or U2OS cells expressing CRM1-HA were synchronized in mitosis and infected with Ad5 particles for 30 min, 1 h, or 2 h in the absence or presence (+) of LMB. Cells were fixed and subjected to indirect immunofluorescence using antibodies against the HA tag (magenta), Ad5 capsids (green), and pVII (red). DAPI (gray) was used for chromatin staining. (C) Confocal images of cells after 1 h of infection. Scale bars,10 μm. (D and E) Quantification of the results shown in panel C. Scatterplots show the number of pVII foci normalized to the number of Ad5 capsids per cell. (D) Comparison of U2OS cells not treated with LMB (black) and U2OS cells expressing CRM1 C528S-HA and treated with LMB (gray). (E) Comparison of U2OS cells expressing CRM1 C528S-HA (gray) or CRM1 W142A P143A C528S-HA (blue), both treated with LMB. Mean values (±SD) of 30 cells per condition are shown. Statistical analysis was performed using a one-way ANOVA multicomparison test.

As unexpected as capsid disassembly and genome release were for mitotic cells, our results clearly showed that the addition of LMB impaired the process. There is no compartmentalization between cytoplasmic and nuclear factors in mitotic cells, and CRM1 cargos should be readily available. It is unlikely that LMB cargo sequestration was responsible for the disassembly defect, favoring a direct and nuclear export-independent role for CRM1. We thus repeated the rescue experiment presented in Fig. 3 using overexpression of a range of LMB-resistant CRM1 mutants. U2OS cells were transfected with HA-tagged and LMB-resistant CRM1, arrested in mitosis, and infected with fluorescently labeled Ad5 (Fig. 8A). Infections were carried out in the presence of LMB (except in control cells). At 1 h p.i., cells were fixed and the efficiency of capsid disassembly in mitotic cells expressing HA-tagged CRM1 was quantified according to the number of pVII dots normalized to the total number of Ad5 capsids per cell (Fig. 8B). Under these conditions, capsid disassembly was severely impaired in the presence of LMB but was fully rescued when a cell expressed CRM1 C528S (Fig. 8B). Similar to the results obtained in interphase cells, only the CRM1 W142A P143A C528S triple mutant was unable to rescue capsid disassembly and genome release. This was especially striking when individual cells were compared (Fig. 8B). Mitotic cells expressing CRM1 C528S reproducibly produced higher levels of pVII dots after 1 h of infection than untreated control cells. We thus decided to compare the kinetics of capsid disassembly and genome release in mitotic cells relying on either endogenous CRM1 (without LMB treatment) or overexpressed CRM1 C528S (with LMB) (Fig. 8C). The results showed accelerated capsid disassembly in cells overexpressing CRM1 compared to that of cells relying on endogenous CRM1, while the overall disassembly rate resumed at 2 h p.i. (Fig. 8D). In contrast, the CRM1 W142A P143A C528S triple mutant was severely impaired (Fig. 8E). Taken together, our experiments provide strong evidence that CRM1 directly affects capsid disassembly and that this function is independent of nuclear export. Furthermore, our results suggest that CRM1 availability is rate limiting for this process.

## DISCUSSION

### A CRM1 mutant defective in capsid disassembly.

In this study, we dissected the different steps of the Ad5 nuclear transport process, starting with accumulation of viral capsids at the MTOC. We confirmed that Ad5 can enter enucleated cells, resulting in virus accumulation at the MTOC, similar to what is observed with LMB treatment. Using a galectin-3 puncta assay (14), we showed that Ad5 enters the cytosol of enucleated cells by endosomal escape. This suggests that particles accumulating at the MTOC have gone through a partial disassembly process following endosomal uptake, corresponding to the natural infection route, and do not accumulate through artificial membrane openings inflicted during enucleation. Hence, our assay confirms that for the first part of the journey, Ad5 does not rely on intact nuclei or nuclear factors like CRM1, which was largely removed from enucleated cells.

In contrast, the LMB block at the MTOC could be overcome by introducing an LMB-resistant mutant of CRM1. Using a small-scale screen of CRM1 mutants on the LMB-resistant background (C528S), we identified the CRM1 W142A P143A C528S triple mutant to be defective in rescuing capsid transfer from the MTOC to the NPC and genome import. Importantly, the mutant, which showed only a negligible reduction in affinity to NES peptides, was able to transport nuclear export substrates at normal levels *in vivo* and in an *in vitro* export assay. Furthermore, in the presence of LMB, stable cells relying on this triple mutant grew at a similar rate as cells expressing the CRM1 C528S single mutant. These observations showed that whatever the effect the mutant has on Ad5, it is not directly linked to the canonical nuclear export function of CRM1. This is an important finding, as it argues against the possibility that CRM1 simply exports an essential factor to the cytoplasm that then alleviates the MTOC arrest under LMB treatment. It is also corroborated by our results in mitotic cells (see below), where LMB affected capsid disassembly in the absence of an intact nucleus. We consider three related alternatives to an indirect CRM1 effect through export of an unknown factor: first, CRM1 alone could directly bind to the viral capsid or any other exposed core protein and initiate disassembly. Second, CRM1 could act in a complex with RanGTP, which is expected to have a higher concentration in the immediate vicinity of the NPC than in the rest of the cytoplasm. Binding to an exposed NES on the viral capsid would elicit the observed effects. The NES cleft of CRM1, however, is opposite to the crucial amino acid residues 142 and 143 (Fig. 4A), arguing against this possibility. Third, binding of CRM1 to the viral capsid could be promoted by FG repeats of a relevant nucleoporin, e.g., Nup214. A related observation has recently been made in the yeast Schizosaccharomyces pombe (65). There, the FG repeats of the Nup214 homologue Nup146 promote CRM1- and RanGTP-dependent docking of Mto1/2 to the NPC. Mto1 is clearly not a nuclear export cargo, yet its binding to CRM1 requires an NES-like sequence. Interestingly, Mto1/2 recruit γ-tubulin to MTOCs and determine where they are generated in the cytoplasm (66). These models have in common that they invoke a role of CRM1 in the disassembly of viral capsids that is functionally distinct from its canonical role in nuclear protein export. So far, we (and others [38]) did not succeed in showing such direct interactions of CRM1 with the capsid (with or without Ran or FG repeats), neither with purified factors nor using pulldown experiments from cells. Thus, a so far unknown cellular factor might promote such an interaction. Importantly, we analyzed capsid localization and genome import over time and showed that the triple mutant strongly delays but does not abolish Ad5 trafficking and genome delivery. Once genomes were delivered, E1A gene expression resumed and overall viral infectivity was not impaired. Hence, the CRM1 function that is compromised in the triple mutant is probably required for the viral entry process only.

### Capsid disassembly in mitotic cells.

Capsid translocation from the MTOC and genome import are the consequences of several discrete steps, including microtubule-dependent transport, binding and removal from the microtubules, docking at the nuclear pore, capsid disassembly, and genome release, followed by nuclear import of the released genome (56). It is unlikely that CRM1 controls all of these steps. Here, we exploited mitotic cells to simplify and dissect the process. The current view is that viral capsid disassembly and genome release occur at the NE, following docking and mechanical capsid disruption at the NPC (23, 26, 28, 30), and that these steps are required for genome delivery into the nucleus. Infection of mitotic cells previously showed a CRM1-dependent association of Ads with spindle microtubules, but no genome release was observed (37). Strunze et al. used thymidine treatment, which preserves microtubules and spindle poles. With the Colcemid treatment used in our study, microtubule polymerization is impaired, leading to a defective assembly of the spindle poles by the time we observed mitotic cells. Consequently, the addition of LMB did not result in strong accumulation at the spindle poles. Remarkably, under these conditions, we were able to follow the release of the viral genomes from the capsid and its chromatin association in living and fixed cells. These observations showed that mitotic cells can indeed be infected by adenovirus, putting into question the strictness of the current view described above. We followed mitotic infections only for a short period of time. To estimate the biological impact of our observation, it remains to be addressed if mitotic infection is indeed productive.

To our surprise, we observed a clear LMB effect in mitotic cells. Instead of having a gross effect on capsid localization, LMB inhibited genome release. This could be restored, however, by overexpression of LMB-resistant CRM1 wild type but not by the CRM1 W142A P143A C528S triple mutant. This indicated an unexpected role for CRM1 in capsid disassembly. For cells in interphase, adenovirus capsid disassembly and genome release are currently believed to take place at the NPC (23, 28). It was proposed that disassembly requires the interaction of the intact capsid via the hexon protein with the nucleoporin Nup214, while the binding of anterograde motors to capsid surface-exposed protein IX induces disassembly (28). We recently showed that protein IX-deficient capsids are efficient in nuclear genome delivery (26), suggesting that alternative mechanisms are involved in capsid disassembly. Recently, it was shown that the ubiquitin ligase Mib1 and the proteasome also contribute to genome release at the NPC (67, 68). The mechanisms are still unclear and may involve degradation of the genome-associated viral protein V (69). Our observation that capsids also disassemble in mitotic cells, i.e., in the absence of an intact NE or NPC, indicates that they do not necessarily require anchoring at the NPC for disassembly, although we cannot exclude the possibility that residual microtubules contribute to capsid disassembly in our system. Nup214 is present and accessible in mitotic cells, where NPC subcomplexes are retained (i.e., Nup214-p62) (70). As suggested for Nup146 and Mto1 (65), FG repeats of such complexes, together with CRM1, could act on viral capsids in mitotic cells, functioning as NPC surrogates. However, the affected domain in the CRM1 triple mutant is unlikely to directly participate in Nup214 binding (Fig. 4), suggesting that its role in promoting capsid disassembly is more complex.

CRM1 and Ran also participate in the biogenesis of centrosomes (65, 71, 72). Moreover, a mutagenesis approach showed that the N-terminal 112 amino acid residues of CRM1 are required to target the protein to the MTOC (54). While we failed to observe CRM1 at the MTOC, it is possible that CRM1 plays a role in the removal of Ad5 from the MTOC and the transfer to the NE in interphase cells (or displacement from a bound factor in mitotic cells) to promote capsid disassembly, e.g., via Mib1 and the proteasome.

Together, our data establish CRM1 with a conserved domain as an essential factor for capsid disassembly and genome release in interphase and also in mitotic cells. How CRM1 fulfills this function remains to be investigated.

## MATERIALS AND METHODS

### Plasmids.

The original plasmids coding for CRM1-HA and the C528S mutant were described previously (73). Additional mutations (W142A P143A, W880A F882A, P967 V968A, P778A P779A, and D824K W880A) were introduced by site-directed mutagenesis. A DNA fragment carrying the mutations L679R K680Q and A731Q was synthesized by GeneArt (Thermo Fisher) and cloned into the parent vector coding for CRM1-HA. Site-directed mutagenesis (74) finally yielded CRM1-HA-C528A D824K L679R K680Q A731Q. The plasmid coding for enhanced GFP (EGFP)-SPN1 was described previously (75). A His-HA-tagged version of human CRM1 wild type was generated from pET21a-CRM1-His (kindly provided by R. Ficner), using primers 5′-AAATGGGTCGCGGATCCATGCCTGCAATTATGACC and 5′-GCACTCGAGTTAAGCGTAATCTGGAACATCGTATGGGTAGTGATGGTGATGGTGATG (underlined nucleotides code for the HA tag). For site-directed mutagenesis (74), oligonucleotides 5′-GACCTGCTGGGTCTGAGTGAACAGAAACGTGGT and 5′-ACCACGTTTCTGTTCACTCAGACCCAGCAGGTC and 5′-CAGATTCTGAAAC AAGAAGCGGCGAAACATTGGCCGACCTTTA and 5′-TAAAGGTCGGCCAATGTTTCGCCGCTTCTTGTTTCAGAATCTG were used, yielding the CRM1 C528S and CRM1 W142A P143A C528S mutants, respectively. Plasmid pCAG-H2BtdiRFP-IP was a gift from Maria-Elena Torres-Padilla (Addgene plasmid no. 47884) (76).

### Cell culture.

U2OS cells (ATCC HTB-96; kindly provided by M. Piechaczyk, IGMM, Montpellier, France), U2OS gal3 mCherry (15), U2OS-TAF-I (61), HEK-293 αvβ5 cells (based on ATCC CRL-1573; kindly provided by G. Nemerow, Scripps Research Institute, La Jolla, CA, USA), and HeLa cells expressing GFP-NFAT (41) were grown in Dulbecco’s modified Eagle Medium (DMEM; Life Technologies), supplemented with 100 U/mL of penicillin, 100 μg/mL of streptomycin (Life Technologies), and 10% fetal calf serum (Life Technologies). All cell lines were regularly screened for the absence of mycoplasma. Cells were maintained in a humidified incubator at 37°C, with 5% CO_2_. For LMB treatment, cells were incubated with 20 nM LMB (Sigma) for 45 min at 37°C. For generation of U2OS cells stably expressing CRM1-HA, cells were transfected with 2 μg of plasmids coding for CRM1 C528S-HA or CRM1 W142A P143A C528S-HA, using Lipofectamine 2000 (Life Technologies). At 48 h posttransfection, 20 nM LMB was added to fresh DMEM for selection. Surviving cells were further maintained in DMEM supplemented with 2 nM LMB. For growth analysis, 2 × 10^5^ cells were initially plated in wells of a 6-well plate and counted after 3, 6, and 9 days using the CASY counting system (Schärfe system). The initial cell number was normalized to 100, and subsequent counts were normalized accordingly. For transient expression, 0.5 to 2 μg of plasmids was transfected using Lipofectamine 2000. To synchronize cells in mitosis, cells were grown on coverslips precoated with 0.01% of poly-l-lysine (poly-l-lysine solution, 0.1% [wt/vol]; Sigma) and treated for 16 h with 0.04 μg/mL of Colcemid (Sigma). Colcemid was included during LMB treatment and replaced with fresh DMEM with LMB but without Colcemid upon Ad5 infection.

### Enucleation of cells.

The protocol for enucleation of cells is based on a previous publication (57) and was optimized for U2OS cells. A total of 3 × 10^5^ U2OS or U2OS gal3-mCherry cells were seeded in a dish (μ-Dish, 35 mm, low; Ibidi) in a total volume of 1 mL of DMEM. The day after, cells were washed once with phosphate-buffered saline (PBS), and 1 mL of DMEM containing 10 μg/mL of cytochalasin B (Enzo Life Sciences) was added. After incubation for 45 min at 37°C, fresh medium containing 10 μg/mL of cytochalasin B was added to entirely fill the dish with liquid. The dishes were placed upside-down in centrifuge bottles of 250 mL filled with paper to wedge the dishes horizontally. The cells were centrifuged in a Sorvall GSA rotor at 11,000 rpm for 50 min at room temperature (RT). After centrifugation, cells were washed three times with PBS and incubated at 37°C with DMEM for at least 90 min before infection.

### Ad5 production and infection protocols.

Ad5 vectors used in this study are based on the genotype of HAdV-C5 with the wildtype (wt) sequence containing a deletion of the E1/E3 region expressing GFP under a cytomegalovirus (CMV) promoter from the E1 region (i.e., Ad5-wt-GFP) or with a deletion only for the E3 region for its replication-competent counterpart with an intact E1 region (i.e. Ad5-wt, used for the RNAScope experiment). Virus amplification was done in E1-complementing HEK-293 αvβ5 cells, and virus particles were purified from infected cells using double CsCl_2_ banding (6, 77). Ad5 physical particles were quantified in accordance with a previously published method (78). Viral particles were fluorescently labeled using an Alexa Fluor microscale labeling kit (Life Technologies) (59). Cells were infected with ∼3,000 physical particles per cell. The viral inoculum was supplemented or not with 20 nM LMB and added to cells for 30 min at 37°C. After 30 min, the inoculum was removed and replaced with fresh DMEM (with or without LMB) to synchronize infections. Inoculum removal was considered the starting point of the infection. For infections of mitotic cells, the 30-min incubation was performed on ice.

For microtubule depolymerization experiments, cells were infected for 2 h as described above, washed with PBS, and either incubated for 30 min at 4°C prior to fixation for cold depolymerization or incubated with 0.1% of digitonin diluted in transport buffer [TPB; 20 mM HEPES (pH 7.3), 110 mM potassium acetate (KOAc), 2 mM Mg(OAc)_2_, 1 mM EGTA; supplemented with protease inhibitor) for 5 min at 37°C prior to fixation, for cell permeabilization.

### Live-cell imaging.

U2OS-TAF-I cells were transfected with 2 μg of pCAG-H2BtdiRFP-IP using Lipofectamine 2000. After 24 h of transfection, cells were detached with 0.05% trypsin/EDTA (Gibco trypsin/EDTA, 0.25% [1×]; Sigma) and seeded on imaging Ibidi μ-slides. After cell attachment, fresh DMEM medium containing 40 μg/mL of Colcemid was added to the cells for 16 h at 37°C. Cells were then washed three times with CO_2_-independent imaging medium (ThermoScientific) in the absence or presence of 20 nM LMB. Infections with Ad5 were performed with fluorescently labeled Ad5-wt-GFP diluted in imaging medium (with or without LMB), with ∼3,000 physical particles per cell without subsequent inoculum removal. Live-cell imaging was performed on a spinning-disk LIFA microscope (Leica) piloted by MetaMorph, equipped with an environmental chamber and an EMCCD camera (Photometrics Quantum 512). Seven stacks of 0.3 μm were taken every 5 s for each channel using a 100× objective. Images were analyzed with Image J (National Institutes of Health).

### Nuclear export assay in permeabilized cells.

Nuclear export of GFP-NFAT in permeabilized HeLa cells was essentially analyzed as described before (41, 79). Briefly, nuclear import of GFP-NFAT was initiated by the addition of ionomycin to intact cells. Cells were then permeabilized with digitonin and subjected to nuclear export reactions at 30°C in the presence of 1 μM RanGTP and 125 nM recombinant CRM1, in a final volume of 40 μL. Reactions were stopped by the addition of 500 μL of cold TPB, and the residual nuclear fluorescence of 10,000 cells was measured by flow cytometry using a FACSCanto II.

### Antibodies.

The following antibodies were used in this study: rabbit anti-HAd5-C5 (serum) (1:1,000; kindly provided by R. Iggo, Institut Bergonie, Bordeaux, France); mouse anti-protein VII (1:100) (61); goat anti-CRM1 (1:500) (80); goat anti-Nup358 (amino acids 2553 to 2838) (81) (1:1,000); rabbit anti-Nup214 (82) (1:1,000); rat anti-HA tag (clone 3F10; Roche) (1:500); rabbit anti-RanBP1 (1:250) and rabbit anti-lamin A/C, dilution 1:200 (kindly provided by L. Gerace, Scripps Research Institute, La Jolla, USA); and mouse anti-tubulin (1:500) (T6199; Sigma-Aldrich) and rabbit anti-pericentrin (ab4448; Abcam) (1:500). For indirect immunofluorescence, cross-adsorbed secondary antibodies from donkey coupled with Alexa Fluor 488, 594, or 647 (Life Technologies) were used at a dilution of 1:500. For Western blotting, primary antibodies were diluted in blocking buffer (25 mM Tris [pH 7.4], 0.137 M NaCl, 2.7 mM KCl, supplemented with 0.05% Tween and 10% milk). Secondary antibodies from donkey coupled with IRDye 680 or 800 (LI-COR) were used at a dilution of 1:10,000 in blocking buffer. Signals were detected with the Odyssey CLx (LI-COR).

### IF staining.

Cells grown on coverslips were washed three times with PBS, followed by 15 min of fixation with 4% paraformaldehyde (PFA; Delta Microscopies) in PBS at RT. Three washes with PBS were performed before cells were processed for immunofluorescence (IF). Cells were permeabilized and blocked with IF buffer (10% fetal calf serum [FCS] and 0.1% saponin in PBS) for 15 min at RT. Primary and secondary antibodies were diluted in IF buffer and added to the cells for 1 h at 37°C in a humidity chamber, with two washes in PBS between each incubation. After the last wash with PBS, coverslips were rinsed with water and 100% ethanol, air dried, and mounted in medium contain either Dako (Agilet) or Mowiol (MOWIOL 4-88; Calbiochem) mixed with 1 μg/mL of DAPI (Sigma).

### Microscopy and image quantifications.

Bright-field fluorescence microscopy was performed using either a Leica DMI6000 B inverted microscope equipped with a sCMOS camera piloted by MetaMorph software or a Nikon Eclipse Ti2 inverted microscope equipped with the NIS-Elements AR 5.02. Confocal imaging was performed using a Leica TCS SP8 and the Leica LAS-X software. The pinhole was set to 1, and z-stacks were collected at 0.3-μm intervals, with 10 planes collected per stack for image analysis. Images were acquired at a 16-bit resolution with a pixel size of 80 nm. For confocal image quantification, channels were split and analyses were performed on z-projections of 10 stacks. The cell periphery was defined manually. A threshold was applied to every channel to select signals of interest. Objects exceeding a size of 5 pixels were considered positive for Ad5 capsids and E1A channels, and a minimal size of 10 pixels was used as a threshold for pVII signals. A minimal size of 500 pixels was determined to identify nuclei. Signals were quantified using a semiautomated macro. For colocalization analyses, signals obtained in two different channels were superimposed, and structures with at least a 5-pixel overlap were considered colocalizing. To quantify the GFP signal in transfected cells, its intensity was measured within the GFP channel, and the ratio between the total and the nuclear (as obtained by superimposition with the DAPI signal) values were plotted. All macros will be made available upon request.

### Statistical analysis.

Image quantifications were performed on a minimum of 30 cells per condition. Results of quantifications are represented as scatterplots with mean values ± standard deviation (SD). Statistical analyses were performed using GraphPad Prism 7 software and a one-way analysis of variance (ANOVA) test. Multicomparison post hoc tests were performed to compare the groups between themselves. Sidak’s post hoc test was used after one-way ANOVA tests. Multicomparison post hoc test results are indicated on the graphs with the following nomenclature: ns, nonsignificant; *, *P* < 0.05; **, *P* < 0.01; ***, *P* < 0.001; ****, *P* < 0.0001.

### RNAScope.

RNAScope assays were performed in accordance with the RNAScope multiplex fluorescent assay adapted protocol (ACD Bio, Newark, CA) (63) and combined with indirect immunofluorescence. Briefly, cells grown on coverslips were fixed with 4% PFA for 10 min at RT, washed with PBS, and incubated successively for 5 min with 50%, 70%, and 100% ethanol. Cells were then incubated for 2 min each with 70% ethanol and 50% ethanol. After incubation for 10 min in PBS, 100 μl of protease III (provided in the kit) diluted 1:30 in PBS was added to the cells, and incubation was continued for 15 min at RT. After three washing steps with PBS, hybridization with the E1A probe (ACD probe no. 497899) was performed for 2 h at 40°C in a humidified chamber. Cells were then washed twice for 2 min with the provided wash buffer and hybridized with the “amplifiers,” in accordance with the manufacturer's instructions. Samples were washed twice with PBS and subjected to indirect immunofluorescence as described above.

### Purification of proteins.

RanQ69L used for *in vitro* nuclear export assays and RanQ69L 1–180 used for anisotropy assays were purified as described previously (83, 84). The expression and purification of His-HA-tagged CRM1 was adapted from a published protocol (85). Briefly, CRM1 was expressed in Escherichia coli BL21(DE3) grown in 2YT medium. Protein expression was induced at an optical density at 600 nm (OD_600_) of 0.5 by the addition of 100 μM isopropyl-β-d-1-thiogalactopyranoside (IPTG). Cells were grown overnight at 18°C, harvested, and frozen at −80°C. Bacterial pellets were resuspended in lysis buffer (50 mM HEPES [pH 7.8], 500 mM NaCl, 2 mM MgCl_2_, 30 mM imidazole, 10% glycerol, and 4 mM β-mercaptoethanol freshly supplemented with 1 mM phenylmethylsulfonyl fluoride [PMSF] and 1 μg/mL each of aprotinin, leupeptin, and pepstatin), and lysis was performed using an Emusiflex C3 emulsifier (Avestin). Lysates were cleared at 30,000 × *g* for 45 min at 4°C and incubated with Ni-nitrilotriacetic acid (NTA) agarose beads (Qiagen) equilibrated in lysis buffer for 90 min at 4°C. Proteins were eluted with elution buffer (50 mM HEPES [pH 7.8], 500 mM NaCl, 4 mM MgCl_2_, 400 mM imidazole, and 3 mM β-mercaptoethanol freshly supplemented with 1 mM PMSF and 1 μg/mL each of aprotinin, leupeptin, and pepstatin). After a buffer exchange to 50 mM HEPES (pH 7.8), 50 mM NaCl, and 4 mM MgCl_2_ supplemented with 2 mM dithiothreitol (DTT) using PD-10 desalting columns (GE Healthcare), proteins were further purified by ion-exchange chromatography using a MonoQ column (GE Healthcare) and an elution gradient of 0 to 250 mM NaCl. Fractions containing CRM1 were pooled, the buffer was exchanged to TPB [20 mM HEPES, 110 mM KOAc, 2 mM Mg(OAc)_2_, 1 mM EGTA, pH 7.3, 1 mM DTT] using PD-10 desalting columns. After concentration using Amicon UltraCel-50K filters (Merck), proteins were frozen in liquid nitrogen and stored at −80°C.

### Anisotropy assays.

Fluorescence polarization assays were performed in anisotropy buffer [20 mM Tris HCl (pH 7.4), 50 mM NaCl, 1 mM Mg(OAc)_2_, 0.005% digitonin, and 2 mM DTT] as described previously (62), using a fluorescent NES peptide derived from the heat-stable inhibitor of cAMP-dependent protein kinase (PKI). Briefly, 40 nM NES peptide labeled with 6-carboxyfluorescein was mixed with increasing concentrations of CRM1-His-HA and 3 μM RanQ69L 1–180 loaded with GTP in a final volume of 150 μL. A 6 μM concentration of the Ran mutant was used when the concentration of CRM1 was higher than 1 μM. Reaction mixtures were incubated at 25°C for 30 min in the dark, and the ratio of polarized light emitted by the fluorophore to the total light intensity was measured using a FluoroMax-4 spectrofluorometer (Horiba, Kyoto, Japan).

### Computational analysis of mutation.

ConSurf analysis (86) of the proteins from the Nup214-CRM1 export complex crystal structure (PDB ID 5DIS) was performed on the ConSurf webserver (https://consurf.tau.ac.il). The multiple sequence alignment was built using MAFFT with 150 homologues collected from UNIREF90. The search was performed using HMMER with one iteration and an E value of 0.0001. Conversation scores were calculated with the Bayesian method. Images were prepared in UCSF Chimera version 1.14, and the figure was assembled using Affinity Designer version 1.7.3.
