# Supplementary material for: CRM1 Promotes Capsid Disassembly and Nuclear Envelope Translocation of Adenovirus Independently of Its Export Function
Source: J Virol. 2022 Feb 9;96(3):e01273-21. doi: 10.1128/jvi.01273-21 (PMC8826800; doi:10.1128/jvi.01273-21)
Supplement: Supplemental file 4 — Supplemental Movie Legends. Download jvi.01273-21-s0005.pdf, PDF file, 0.02 MB [file jvi.01273-21-s0005.pdf]

SUPPLEMENTARY DATA

Movie S1

Cells were imaged using live cell microscopy following the enucleation procedure (see methods section for details). To verify cellular integrity following enucleation, the medium was supplemented with fluorescently labeled antibodies to provide fluid phase contrast. Intact cells exclude the fluorescence. Cells containing a nucleus (magenta arrows) and enucleated cells (cyan arrows) are indicated. Note that enucleated cells are very flat by comparison.

Movies S2-S4

(see also legend to figure 7)

Mitotic U2OS-TAF1β-GFP cells transfected with tdi-RFP-labeled H2B and infected with fluorescently labeled Ad5 and treated with vehicle control (movie S2) or with LMB (movie S3) were recorded using live-cell microscopy. Movies showing infected cells started ~120 min pi and lasted for 17 min. Mitotic, non-infected and untreated U2OS-TAF1β-GFP cells (movie S4) were used as control. Seven stacks of 0.3 µm each around the midsection were taken every 5 sec for each channel using a 100X objective. Merged Z-projections of each channel are shown.
